# Synergistic Anticandidal Effectiveness of Greenly Synthesized Zinc Oxide Nanoparticles with Antifungal Agents against Nosocomial Candidal Pathogens

**DOI:** 10.3390/microorganisms11081957

**Published:** 2023-07-31

**Authors:** Mohamed Taha Yassin, Fatimah O. Al-Otibi, Abdulaziz A. Al-Askar, Marwa M. Elmaghrabi

**Affiliations:** Botany and Microbiology Department, College of Science, King Saud University, Riyadh 11451, Saudi Arabia; falotibi@ksu.edu.sa (F.O.A.-O.); aalaskara@ksu.edu.sa (A.A.A.-A.); 443203278@student.ksu.edu.sa (M.M.E.)

**Keywords:** green synthesis, *Salvia officinalis*, zinc oxide nanoparticles, antifungal, synergism

## Abstract

The high prevalence of fungal resistance to antifungal drugs necessitates finding new antifungal combinations to boost the antifungal bioactivity of these agents. Hence, the aim of the present investigation was to greenly synthesize zinc oxide nanoparticles (ZnO-NPs) using an aqueous leaf extract of *Salvia officinalis* and investigate their antifungal activity and synergistic efficiency with common antifungal agents. The biofabricated ZnO-NPs were characterized to detect their physicochemical properties. A disk diffusion assay was employed to investigate the antifungal effectiveness of the greenly synthesized ZnO-NPs and evaluate their synergistic patterns with common antifungal agents. The *Candida tropicalis* strain was detected to be the most susceptible strain to ZnO-NPs at both tested concentrations of 50 and 100 µg/disk, demonstrating relative suppressive zones of 19.68 ± 0.32 and 23.17 ± 0.45 mm, respectively. The minimum inhibitory concentration (MIC) of ZnO-NPs against the *C. tropicalis* strain was 40 µg/mL, whereas the minimum fungicidal concentration (MFC) was found to be 80 µg/mL. The highest synergistic efficiency of the biogenic ZnO-NPs with terbinafine antifungal agent was detected against the *C. glabrata* strain, whereas the highest synergistic efficiency was detected with fluconazole against the *C. albicans* strain, demonstrating relative increases in fold of inhibition area (IFA) values of 6.82 and 1.63, respectively. Moreover, potential synergistic efficiency was detected with the nystatin antifungal agent against the *C. tropicalis* strain with a relative IFA value of 1.06. The scanning electron microscopy (SEM) analysis affirmed the morphological deformations of candidal cells treated with the biosynthesized ZnO-NPs as the formation of abnormal infoldings of the cell wall and membranes and also the formation of pores in the cell wall and membranes, which might lead to the leakage of intracellular constituents. In conclusion, the potential synergistic efficiency of the biogenic ZnO-NPs with terbinafine, nystatin, and fluconazole against the tested candidal strains highlights the potential application of these combinations in formulating novel antifungal agents of high antimicrobial efficiency. The biogenic ZnO nanoparticles and antifungal drugs exhibit powerful synergistic efficiency, which highlights their prospective use in the formulation of efficient antimicrobial medications, including mouthwash, ointments, lotions, and creams for effective candidiasis treatment.

## 1. Introduction

According to a prior report, fungal diseases have a significant impact on a global scale, resulting in the deaths of over 1.5 million individuals and affecting more than a billion people annually [1]. According to recent literature, it is estimated that over 300 million individuals suffer from a fungal infection, which leads to more than 1,350,000 fatalities each year [2]. *Candida* is presently regarded as the fourth most prominent cause of nosocomial infection, particularly in patients with compromised immune systems, with a mortality rate ranging from 35% to 100% [3]. *Candida* urinary tract infection is a prevalent nosocomial infection that impacts the urinary tract and is responsible for a minimum of 10–15% of hospital-acquired urinary tract infections [4]. Candiduria is the predominant manifestation of candidiasis observed in patients who have been admitted to the intensive care unit (ICU) [5]. The prevalence of this condition is primarily observed among the elderly population [6]. The detection of *Candida* spp. in urine is frequently linked to elevated mortality rates, particularly among patients in the intensive care unit who have multiple comorbidities. Moreover, *Candida* has the ability to infiltrate the bloodstream, propagate, and result in severe infection, resulting in a medical condition known as candidemia. These infections are frequently linked to severe outcomes, extended hospital stays, and elevated medical expenses [7]. Moreover, candidemia was identified as the predominant cause of healthcare-associated bloodstream infections in a sample of 183 hospitals in the United States [8]. The incidence of Candidemia is often linked to significant mortality rates, which can exceed 40%, despite the emergence of novel antifungal medications [9]. The presence of *Candida* in the bloodstream has the potential to generate novel biofilms. The dissemination of cells within the biofilm to various tissues throughout the body holds significant clinical implications. Candidemia is frequently regarded as the most perilous manifestation of candidiasis. This phenomenon can result in the development of numerous systemic diseases that are widely spread throughout the body. Candidemia continues to be a significant contributor to morbidity and mortality within healthcare environments [10]. *Candida albicans* is primarily accountable for the majority of candidiasis occurrences. Non-albicans species, particularly *C. glabrata*, *C. krusei*, and *C. tropicalis*, have frequently been associated with this phenomenon, as reported by various studies [11,12]. Given the high mortality rate linked to bloodstream infections caused by *Candida* spp., high resistance is concerning [13]. Despite the availability of antifungal medications for Candida infections, the mortality rates associated with this condition remain elevated [14]. The employment of drug categories, including azoles, has resulted in amplified resistance of *Candida* owing to their widespread and prolonged usage [15]. To enhance treatment outcomes in the face of rising resistance, new antifungal medication formulations, combination therapy, and innovative biomaterial formulations are required [16]. Nanotechnology is a dynamic area of scientific and technological inquiry that centers on the synthesis and advancement of nanomaterials with particle dimensions ranging from 1 to 100 nm [17,18]. The unique physicochemical properties of nanoparticles have garnered increased attention from the scientific community [19]. Among the various types of nanoparticles, zinc oxide nanoparticles (ZnO-NPs) hold considerable importance due to their extensive range of applications, which encompass photocatalysis, antimicrobial properties, and water purification [20]. Moreover, ZnO-NPs possess a significant band gap of 3.37 eV, enabling them to efficiently generate electron–hole pairs upon exposure to ultraviolet (UV) radiation. This characteristic renders them suitable for application in electronic devices, as well as for their utilization as photocatalysts in the degradation of textile waste and the removal of heavy metal ions commonly found in industrial settings [21]. The antimicrobial properties of ZnO-NPs against pathogenic fungi and bacteria can be attributed to alterations in cell permeability upon interaction with the cell membrane of microbial cells [22]. The movement of ZnO nanoparticles into the cytoplasm disrupts cellular processes, leading to the creation of a zone of inhibition against microbes [23]. Moreover, ZnO-NPs have been found to induce detrimental effects on the cell membrane, ultimately leading to bacterial cell death [24]. The phenomenon described can be elucidated by the mechanism by which oxygen species are emitted at the surface of nanoparticles, subsequently engaging in a reaction with hydrogen to generate hydrogen peroxide. The hydrogen peroxide produced either inhibits microbial growth or induces bactericidal effects [25]. Recent research has demonstrated that the antimicrobial effectiveness of nanoparticles is significantly enhanced when their size ranges from 1 to 10 nm, owing to the larger surface area available for interaction with microbes [26]. The antimicrobial properties of nanoparticles are currently being extensively researched, including those with microbicidal properties, such as silver, zinc oxide, copper oxide, zero-valent iron, carbon nanotubes, titanium dioxide, and bio-nanoparticles such as chitosan nanocomposites [27]. These nanoparticles exhibit remarkable biocidal properties against various microorganisms, while the possibility of the emergence of resistant strains is not a concern [28]. Physical, chemical, and green processes are used to create nanoparticles [29]. The physical approach uses expensive machinery, operates at high pressure and temperature, and requires a large area for machine setup [30]. Using toxic chemicals, which may be hazardous for the environment and the person handling them, is one of the main drawbacks of the chemical method of synthesis [31]. In contrast, the term “green synthesis” or “biosynthesis” refers to the biological process of the formation of ZnO-NPs, which uses microorganisms and plant extracts as the reducing agents [32]. The biogenic nanoparticles were reported to possess antimicrobial, anticancer, and antioxidant properties [33]. Despite the advantages of utilizing microorganisms as a reducing agent in the production of ZnO nanoparticles, there are significant concerns regarding safety due to the toxicity of specific microbes and challenges associated with incubation [34]. The high efficiency of plant extracts in ZnO-NP biosynthesis has been attributed to the high amount of phytochemicals or secondary metabolites [35]. There have been reports of phytochemicals acting as effective reducing agents for zinc precursors, including methylxanthines, terpenoids, flavonoids, tannins, alkaloids, phenolic acids, and saponins [36]. In addition to being safe, affordable, environmentally friendly, non-hazardous, and biocompatible, this usage of plant extract offers the potential for large-scale production [37]. A prior report indicated the anticandidal efficiency of ZnO-NPs fabricated utilizing *Girardinia diversifolia* extract against *C. albicans* strains with a relative suppressive zone diameter of 20.23  ±  0.65 mm [38]. Moreover, the biogenic ZnO-NPs synthesized using *Carissa opaca* extract revealed antifungal efficiency against *C. albicans* strains with a relative inhibitory zone diameter of 16.0 ± 1 mm [39]. Limited research has been conducted on the combined effectiveness of biogenic ZnO-NPs and antifungal agents against candidal pathogens. Therefore, it is crucial to consistently investigate potential synergistic interactions between biogenic ZnO-NPs and conventional antifungal agents. Moreover, the high prevalence of candidal resistance to antifungal drugs necessitates finding new antifungal agents to boost the antimicrobial effectiveness of antifungal drugs and avoid the possible toxicity of high dosages of antifungal drugs. Hence, the current investigation aimed to greenly synthesize ZnO-NPs utilizing an aqueous leaf extract of *Salvia officinalis*, characterize these nanoparticles, and evaluate their antifungal efficiency against three candidal strains. Moreover, the synergistic proficiency of ZnO-NPs with antifungal agents was also evaluated.

## 2. Materials and Methods

### 2.1. Preparation of S. officinalis Leaf Extract

The dried leaves of *Salvia officinalis* were procured from a local market located in Riyadh, Saudi Arabia. The identification of the plant samples was confirmed by the herbarium of the department of Botany and Microbiology. The dried leaves of *S. officinalis* were subjected to a triple cleaning process using distilled water following a single wash with tap water. Subsequently, they were permitted to completely dry in the open air. The leaves were crushed into a consistent and fine powder using a mechanical blender. A 500 mL flask was utilized to hold a quantity of 50 g of plant powder along with 200 mL of distilled water. The flask was heated at a temperature of 60 °C for 30 min over a hot plate. The flask was then subjected to constant stirring for 24 h at a temperature of 25 °C with the aid of a magnetic stirrer. Subsequently, the mixture was purified using Whatman filter paper (1) to obtain a refined filtrate and eliminate any residual substances. Afterwards, the extract was sterilized via filtration utilizing a 0.45 µm Millipore membrane filter. Finally, the prepared extracts were refrigerated at a temperature of 4 °C for future experiments.

### 2.2. Green Biofabrication of the Biogenic ZnO-NPs

The aqueous extract derived from the leaves of *S. officinalis* was employed in the reduction of zinc nitrate hexahydrate solution for the biopreparation of ZnO-NPs. Zinc nitrate hexahydrate (Zn (NO_3_)_2_·6H_2_O) with a purity of 98% was obtained from Sigma-Aldrich, located in Poole, Dorset, U.K. Five milliliters of *S. officinalis* leaf extract was added to 95 mL of a solution of 0.01 M zinc nitrate hexahydrate. Subsequently, the flask was subjected to magnetic stirring for 60 min at 70 °C. The formation of reduced precipitates is indicative of the formation of ZnO-NPs. The reaction mixture was then centrifuged at 10,000 rpm for 10 min for the separation of the reduced precipitates. In order to eliminate any extract residues, the precipitates were subsequently washed with distilled H_2_O. Finally, the precipitates were incubated in an oven for 8 h at 100 °C for subsequent physicochemical characterization [40].

### 2.3. Physicochemical Characterization of the Biogenic ZnO-NPs

The biosynthesized ZnO-NPs were characterized using different methods, including UV-Vis spectroscopy to determine the surface plasmon resonance of ZnO-NPs. The biosynthesized ZnO-NPs were analyzed for their shape and particle size distribution using transmission electron microscopy (TEM) (JEOL, JEM1011, Tokyo, Japan). Additionally, the elemental analysis of ZnO-NPs was carried out using Energy-Dispersive X-ray (EDX) analysis, while Fourier transform infrared spectroscopy (FTIR) analysis was employed to identify the main functional groups of the biofabricated ZnO-NPs. The biosynthesized ZnO-NPs were subjected to X-ray powder diffraction (XRD) investigation in order to verify their crystalline nature and determine their crystalline size. The surface charge and hydrodynamic diameter of ZnO-NPs were evaluated using a Zeta sizer instrument (Malvern Instruments Ltd.; zs90, Worcestershire, UK).

### 2.4. Screening of Antifungal Effectiveness of ZnO-NPs

The susceptibility of three strains of *Candida*, namely, *C. albicans* (ATCC 29213), *C. glabrata* (ATCC 25922), and *C. tropicalis* (ATCC 33592), to the biogenic ZnO-NPs was evaluated. The antifungal efficacy of ZnO-NPs was assessed using a disk diffusion assay against the tested fungal strains. The preparation of the candidal suspension was performed using sterile saline solution (0.89%). The turbidity of the candidal suspension was then standardized using the 0.5 McFarland standard. Sterile Petri dishes were filled with Mueller–Hinton agar (MHA) medium supplemented with 2% glucose, and the candidal suspension was distributed homogenously over the surface of the MHA plates by using sterile swabs. The dried ZnO-NPs were dispersed in methanol, and then sterile filter paper disks (8 mm in diameter) were saturated with the biogenic ZnO-NPs at concentrations of 50 and 100 µg/disk. Sterile filter paper disks impregnated with 30 µg/disk of Terbinafine antifungal were utilized as positive controls, whereas filter paper disks loaded with methanol only were utilized as negative controls. Successively, the plates were kept in the incubator at 35 °C for 24 h then the Vernier caliper was used to measure the inhibition zone diameters. The minimum inhibitory concentration (MIC) of ZnO-NPs was examined against the tested candidal strains, using a broth microdilution assay in 96-well microtiter plates, as demonstrated in a prior investigation [41]. Moreover, the minimum fungicidal concentration (MFC) was evaluated by streaking the MIC concentration and the two other successive concentrations over freshly prepared MHA plates, followed by incubation at 35 °C for 24 h. The minimum concentration revealing no candidal growth was recorded as MFC.

### 2.5. Detection of Fungal Cell Deformations Utilizing Scanning Electron Microscopy (SEM) Analysis

The morphological deformations of candidal cells treated with the biosynthesized ZnO-NPs were assessed using SEM analysis. Agar fragments were detached from the suppressive zones and subsequently fixed for 60 min at 25 °C in a solution consisting of 3% (*v*/*v*) glutaraldehyde, which was buffered with 0.1 M sodium phosphate buffer at a pH of 7.2. Afterwards, the agar fragments were washed four times in a buffer, and then the fragments were post-fixed in 1% (*w*/*v*) osmium tetroxide (OsO_4_) for 1 h. The samples were then subjected to alcoholic dehydration using ethanol at concentrations ranging from 30 to 100% for 15 min. Following this, the specimens were affixed to stubs using double-sided carbon tape after complete drying. The specimens were coated with a thin layer of gold using a Polaron SC 502 sputter coater. Subsequently, the morphological deformations were investigated using a scanning electron microscope (JEOL JSM-6380 LA).

### 2.6. Evaluation of Synergistic Efficiency of the Biosynthesized ZnO-NPs with Antifungal Agents

The synergistic effectiveness of the biofabricated ZnO-NPs with antifungal agents was evaluated using a disk diffusion assay. In this study, sterile paper disks of 8 mm diameter were loaded with the MIC of the biogenic ZnO-NPs (40 µg/disk). Another set of disks was loaded with MIC concentrations of ZnO-NPs and antifungal agents, including itraconazole, fluconazole, nystatin, terbinafine, and clotrimazole at concentrations of 10 µg, 25 µg, 20 µg, 30 µg, and 10 µg, respectively. Positive control disks were Terbinafine antifungal agents (30 µg/disk), whereas another set of disks were impregnated with methanol as negative controls. Afterwards, seeded MHA plates were prepared, and the impregnated disks were placed over the surface of the seeded plates. Finally, the plates were incubated at 35 °C for 24 h, and then a Vernier caliper was used to assess the diameters of inhibition zones after the incubation period. The increase in fold of inhibition area (IFA) was calculated in accordance with the subsequent equation:

IFA = (B^2^ − A^2^)/A^2^, whereas B and A are the inhibition zone diameters for antifungals + ZnO-NPs and antifungal agents, respectively [42]. Moreover, the fractional inhibitory concentration (FIC) index was estimated using the formula described in previous studies [43,44].

$$\mathrm{FIC}\;\mathrm{index}\;(\mathrm{FICI})=\frac{\mathrm{MIC}\;\mathrm{of}\;\mathrm{drug}\;A\;\mathrm{in}\;\mathrm{the}\;\mathrm{combination}}{\mathrm{MIC}\;\left(A\right)}+\frac{\mathrm{MIC}\;\mathrm{of}\;\mathrm{drug}\;B\;\mathrm{in}\;\mathrm{the}\;\mathrm{combination}}{\mathrm{MIC}\;\left(B\right)}$$

where A refers to the biogenic ZnO-NPs and B refers to antifungal agents. The FICI is detected as follows: synergistic effect ≤0.5; additive 0.5 to 1; no effect >1 to 4 and antagonistic >4.

### 2.7. Cytotoxicity Assay

The methylthiazolyl diphenyl-tetrazolium bromide (MTT) assay was used to assess the cytotoxicity of the biogenic ZnO-NPs synthesized using *S. officinalis* extract against the WI-38 (normal lung fibroblast cells) cell line. The tested concentrations of ZnO-NPs were 25, 50, 100, 200, 400, 800, and 1600 µg/mL. A 96-well tissue culture plate was inoculated with a cell concentration of 1 × 10^5^ cells/mL and a volume of 100 µL per well. The plate was then incubated for 24 h at 37 °C, and then the growth medium was poured off. The biogenic ZnO-NPs were cultured in Roswell Park Memorial Institute (RPMI) medium supplemented with 2% serum and subjected to two-fold dilutions. Each well was subjected to a treatment of 0.1 mL of each dilution, while three wells were designated as controls and received serum only. Subsequently, the plate was subjected to incubation at a temperature of 37 °C for 48 h. The MTT solution, with a concentration of 5 mg/mL in phosphate-buffered saline (PBS), was prepared. Each well was then treated with 8–20 μL of the MTT solution, which was thoroughly mixed for 5 min before being incubated at 37 °C and 5% CO_2_ for 4 h until formazan formation. Subsequently, the formazan was resuspended in 200 μL of dimethyl sulfoxide (DMSO) and subjected to gentle agitation for 5 min. The absorbance at a wavelength of 560 nm was recorded, and the absorbance value associated with the concentration that resulted in a 50% reduction in cell viability (IC_50_) was detected.

### 2.8. Statistical Analysis

The data was analyzed using GraphPad Prism version 8.0 (GraphPad Software, Inc., La Jolla, CA, USA) via the Tukey test in a one-way ANOVA at level 0.05. The experimental procedures were conducted in triplicates, and the resulting data were reported as the mean value of the triplicates ± standard error. OriginPro 2018 was utilized to generate the particle size distribution histogram and XRD pattern.

## 3. Results and Discussion

### 3.1. Green Synthesis of ZnO-NPs

**Figure 1:**
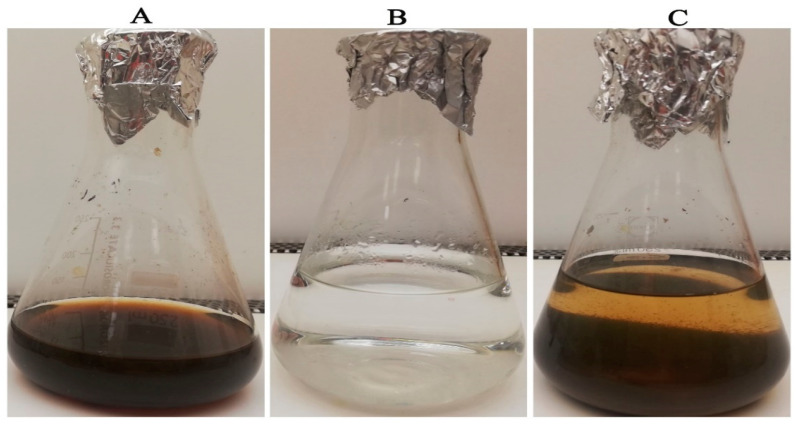
Green synthesis of zinc oxide nanoparticles utilizing water extract of *S. officinalis* (**A**) *S. officinalis* extract, (**B**) Zn(NO_3_)_2_.6H_2_O colorless solution, and (**C**) formation of reddish brown precipitates indicating ZnO-NPs formation.

The green synthesis of ZnO-NPs was conducted using the water extract of *S. officinalis* (Figure 1). The phytochemicals in *S. officinalis* extract reduce the zinc nitrate solution, resulting in the development of a reddish-brown precipitate, affirming the formation of zinc oxide nanoparticles. The phenolic compounds present in plant extracts play a role as both reducing and stabilizing agents in the synthesis of biogenic ZnO-NPs, preventing particle aggregation and providing antioxidant properties [33,45,46]. Previous studies have provided a mechanistic pathway for producing ZnO-NPs based on the chemical constituents of plant extracts. It was hypothesized that the plant extract reduces zinc (II) ions to metallic zinc rather than forming coordinated complexes. After the full reduction of the zinc precursor, a reaction between metallic zinc and dissolved oxygen in the solution occurred, resulting in the production of ZnO nuclei. Moreover, the phtyoconstituents act as a stabilizing agent, preventing particle agglomeration [47].

### 3.2. UV-Vis Spectral Analysis

**Figure 2:**
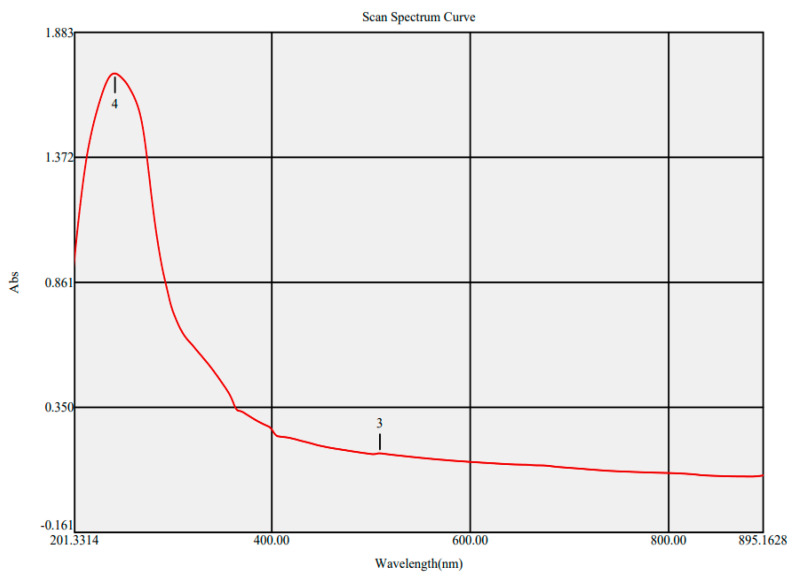
UV spectrum of ZnO-NPs (**4**: 242 nm, **3**: 509 nm).

**Figure 3:**
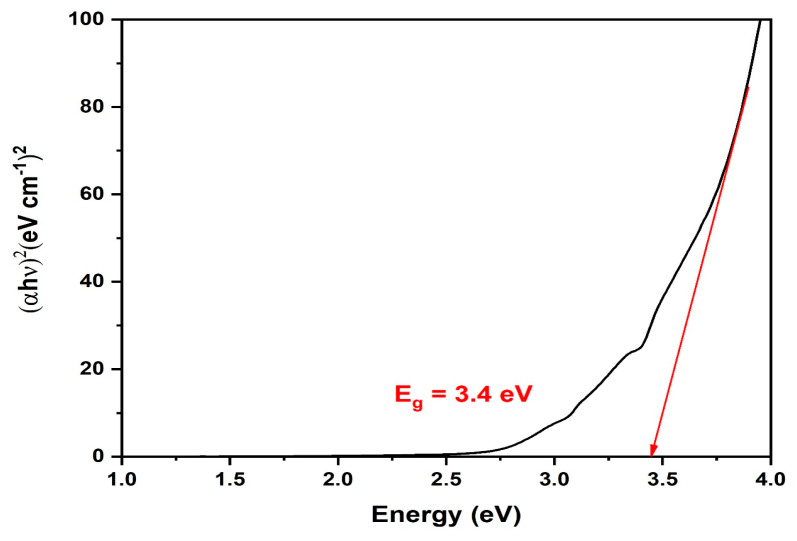
Band gap energy of the biofabricated ZnO-NPs (red arrow was added for determination of the value of band gap energy).

The surface plasmon resonance of the biogenic ZnO-NPs was determined using UV spectral analysis of the bioinspired ZnO-NPs. At 242 and 509 nm, two absorption peaks were found. The peak at 242 nm may be assigned to the phytochemicals employed in the reduction technique, while the distinctive peak at 509 nm could be ascribed to the surface plasmon resonance of ZnO NPs (Figure 2). The observed UV peak with a λ_max_ around 200 nm might be allotted to the absorption of organic molecules onto the ZnO-NPs surface during the reduction procedure. These organic molecules include flavonoids, phenolic acids, and heteroatoms, for example, O, S, N, and unsaturated groups [48]. Our results were in accordance with those of a preceding investigation, which affirmed the presence of a UV band at 509 nm in ZnO-NPs fabricated using peel extract of *Punica granatum* L. [49]. The band gap energy of biogenic ZnO-NPs was estimated using the Tauc plot method and was found to be 3.4 eV (Figure 3). Our finding was in agreement with that of a previous investigation, which reported that the band gap energy of ZnO-NPs formulated using *Coriandrum sativum* leaves was 3.4 eV [50]. Additionally, the biosynthesized ZnO-NPs fabricated using green tea leaf powder indicated that the band gap energy was 3.40 eV, as confirmed by the Tauc plot method [51]. A previous study has demonstrated that the addition of CaO to ZnO in the synthesis of Zn-Ca nanocomposites leads to a decrease in the band gap energy from 3.3 to 2.99 eV [52]. This incorporation leads to only minor fluctuations in the band gap energy. As a result, the addition of donor or acceptor impurities to a semiconductor leads to the formation of energy levels in close proximity to the conduction or valence band edges. Other investigations further explained the factors contributing to the difference in band gap energy of ZnO-NPs, such as calcination temperature, nanoparticle shape, precursors, and induced defects (degree of open lattice structures) [53].

### 3.3. Transmission Electron Microscopy Analysis

**Figure 4:**
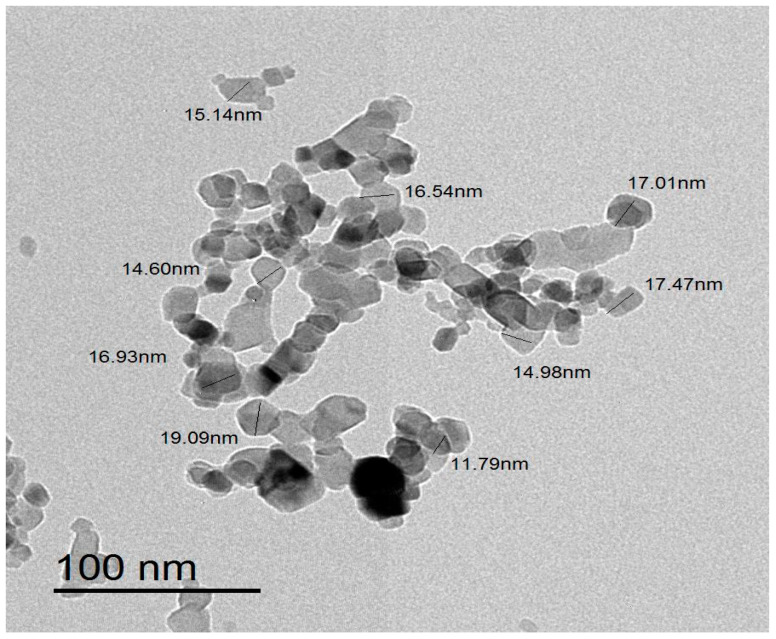
TEM micrograph of the biofabricated ZnO-NPs.

**Figure 5:**
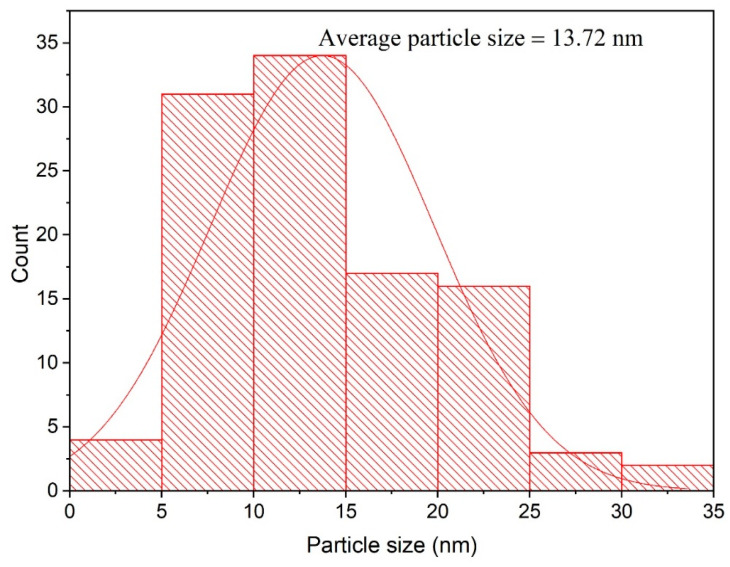
Particle size distribution histogram of ZnO-NPs.

The morphological properties of the biosynthesized ZnO-NPs and the size distribution pattern of the biogenic ZnO-NPs were investigated using TEM analysis. The biogenic ZnO-NPs were hexagonal in form, with particle diameters ranging from 5–35 nm (Figure 4). The average particle size was 13.72 nm, according to the particle size distribution (Figure 5). The small diameter of the produced ZnO-NPs demonstrated the efficiency of the green approach in generating ZnO-NPs of small diameter, which may have potential for use in a variety of applications. The hexagonal structure of ZnO-NPs was affirmed by TEM micrographs. This observation was consistent with a preceding investigation that stated the green biogenesis of hexagonal ZnO-NPs using water extract of *Deverra tortuosa*, as evidenced by TEM micrographs [54]. Moreover, TEM analysis indicated that the average particle size was 13 nm, and this result was in accordance with that of a preceding report, which indicated that the particle size of greenly synthesized ZnO-NPs fabricated utilizing *Azadirachta indica* Gum was 13 nm [55].

### 3.4. EDX Elemental Analysis of the Biogenic ZnO-NPs

**Figure 6:**
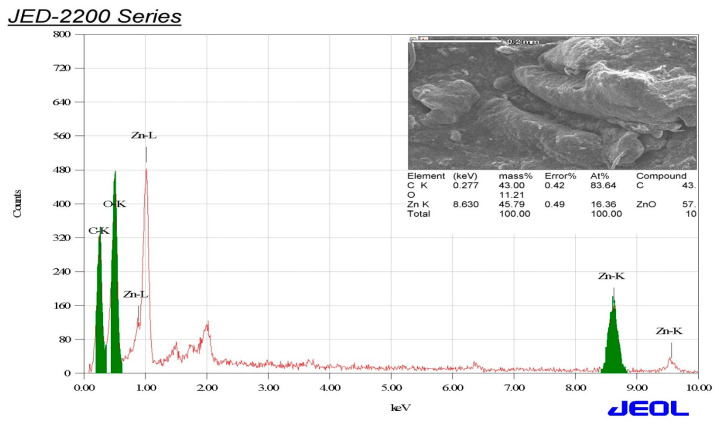
EDX analysis of the bioinspired ZnO-NPs.

EDX analysis was employed to determine the main elements of the biogenic ZnO-NPs. The elemental analysis revealed that carbon, oxygen, and zinc were the main elements of the analyzed sample, with relative mass percentages of 43, 11.21, and 45.79%, respectively. EDX analysis affirmed the successful biosynthesis of ZnO-NPs (Figure 6). The carbon peak was allotted to the carbon tape utilized for placing the biosynthesized ZnO-NPs on the sample holder. The EDX pattern also showed strong peaks at 0.5, 1.1, 8.6, and 9.5 keV, which were allocated to O Kα, Zn Lα, Zn Kα, and Zn Kβ, respectively. These results were in accordance with those of preceding investigations [56,57].

### 3.5. Fourier Transform Infrared Spectroscopy Analysis of the Biogenic ZnO-NPs

**Figure 7:**
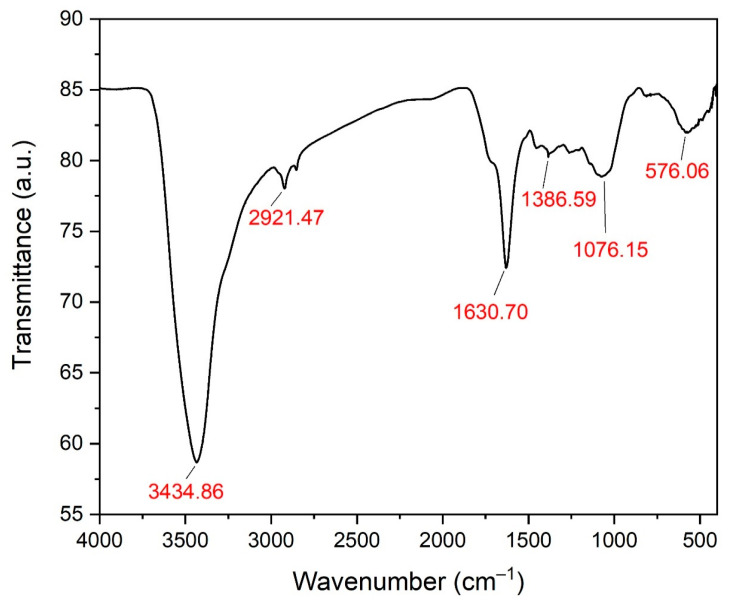
FTIR spectrum of ZnO-NPs.

The functional chemical groups of the biosynthesized ZnO-NPs were investigated using FTIR analysis. The strong band at 3434.86 cm^−1^ might be ascribed to polyphenolic compound O-H stretching [58], while the medium band at 2921.47 cm^−1^ could be related to alkane C-H stretching (Figure 7) [59]. On the other hand, C=C stretching of alkenyl groups was discovered at 1630.70 cm^−1^ [60]. The band at 1386.59 cm^−1^ may be associated with the molecular motion of C-H bending, which may be attributed to the aldehydic group [61]. The median band at 1076.15 cm^−1^ was ascribed to C-O stretching of primary alcohols [62], while the band at 576.06 cm^−1^ indicated the existence of metal oxide bonds (Table 1) [63].

### 3.6. XRD Analysis of the Biogenic ZnO-NPs

**Figure 8:**
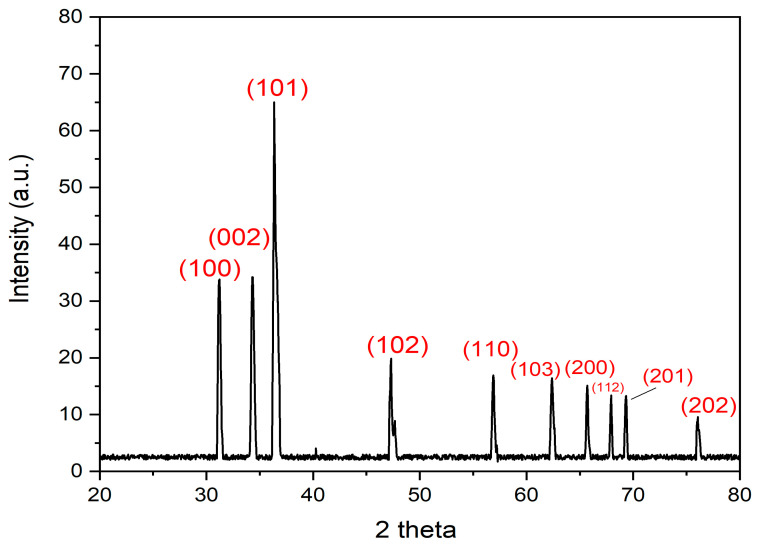
XRD peaks of the bioinspired ZnO-NPs.

XRD analysis revealed the presence of eleven diffraction peaks with 2θ values of 29.54°, 31.14°, 34.28°, 36.45°, 47.25°, 56.83°, 62.51°, 65.62°, 67.91°, 69.32°, and 76.62°. The peaks noticed at 2Ɵ of 31.14°, 34.28°, 36.45°, 47.25°, 56.83°, 62.51°, 65.62°, 67.91°, 69.32°, and 76.62° corresponded to the orientation planes of (100), (002), (101), (102), (110), (103), (200), (112), (201), and (202), respectively (Figure 8). The XRD data affirmed the hexagonal wurtzite structure of ZnO-NPs. The findings of our study were in agreement with those of a prior investigation that demonstrated the production of ZnO-NPs utilizing *Hibiscus rosa*-sinensis extract. The previous study reported X-ray diffraction peaks at 2θ values ranging from 31.73°, 34.38°, 36.22°, 47.50°, 56.56°, 62.81°, 66.34°, 67.91°, 69.03°, 72.6°, and 76.90°, which indicated the crystalline structure of the nanoparticles [64]. Moreover, a previous investigation affirmed this finding and reported the biosynthesis of ZnO-NPs utilizing an aqueous leaf extract of *Lobelia leschenaultiana* with Bragg’s reflection peaks at 100, 002, 101, 102, 110, 103, 200, 201, and 202 planes [65]. The crystalline size of ZnO-NPs was estimated using Scherrer’s formula as follows: L = (kλ/β cosθ), where L is ZnO-NPs crystalline size, λ is the wavelength of X-ray (1.54178 Å), K is Scherer’s constant (K = 0.94), θ is the diffraction angle (36.38°), and β is the full width at half maximum (FWHM) of the most intense diffraction peak that was detected to be 0.4291. The crystalline size was estimated to be 17.74 nm. This result was in agreement with that of a preceding investigation, which revealed the green synthesis of ZnO-NPs utilizing onion extract, recording a crystalline size of 17 nm [66].

### 3.7. Zeta Potential Analysis of the Bioinspired ZnO-NPs

**Figure 9:**
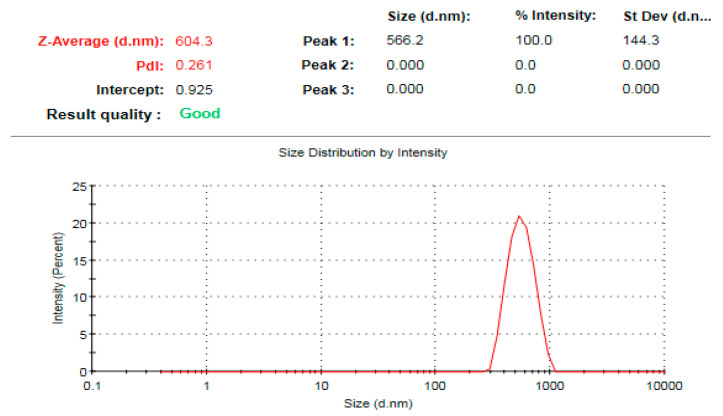
DLS pattern of the biogenic ZnO-NPs.

**Figure 10:**
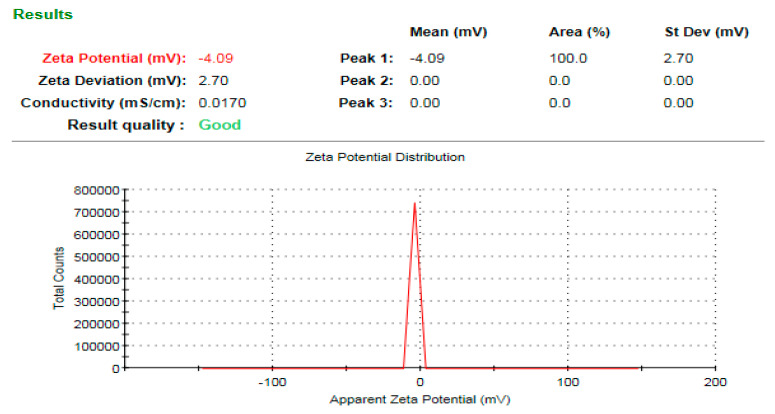
Surface charge of the biofabricated ZnO-NPs.

The average hydrodynamic diameter of ZnO-NPs as determined by dynamic light scattering (DLS) was 604.3 nm, which was significantly higher than that determined by TEM and XRD analysis (Figure 9). This might be attributable to nanoparticle aggregation caused by the action of plant metabolites, as well as the deposition of additional hydrate layers on the surface of the produced nanoparticles, resulting in an increase in hydrodynamic diameter [67,68,69,70]. A previous study confirmed this finding, indicating that the average sizes of DLS are comparatively larger than those obtained from TEM images [71]. This might be due to the fact that DLS measures the particles’ dimensions in three dimensions, which may encompass a biomolecular coating. The ZnO-NPs surface charge was evaluated using zeta potential analysis. The stability of nanoparticles in colloidal systems is dictated by the interparticle interactions, which encompass the electrostatic force, Van der Waals force, and hydrophobic interactions. The determination of electrostatic forces between particles can be deduced by examining the zeta potential value. Consequently, the zeta potential serves as a significant indicator for assessing the stability of Ag-NPs in aqueous suspensions [72]. The biogenic ZnO-NPs had a zeta potential of −4.09 mV (Figure 10). In this approach, the estimated negative charge of ZnO-NPs could be attributable to the *S. officinalis* extract metabolites. The negative charge of the biofabricated ZnO-NPs indicates repulsion between the nanoparticles, which results in high dispersivity and stability. According to a prior study, the presence of adsorbed capping molecules on the surface of colloidal nanoparticles was found to be responsible for their negative charge. This negative charge led to a repulsive force acting between the nanoparticles, thereby hindering their aggregation [73]. Our results were in agreement with those of a preceding investigation, which reported that the average zeta potential analysis of ZnO-NPs was −4.21 mV [74].

### 3.8. Evaluation of Anticandidal Effectiveness of the Bioinspired ZnO-NPs

The effectiveness of the biosynthesized ZnO-NPs against the investigated candidal pathogens was assessed using a disk diffusion technique. *Candida tropicalis* showed the maximum sensitivity to the biofabricated ZnO-NPs at 50 and 100 µg/disk with inhibitory zones of 19.68 ± 0.32 and 23.17 ± 0.45 mm, respectively (Table 2). Moreover, the biofabricated ZnO-NPs (100 µg/disk) exposed antifungal effectiveness against *C. albicans* and *C. glabrata*, demonstrating suppressive zones of 22.56 ± 0.51 and 15.12 ± 0.38 mm, respectively. The fungicidal mechanisms of the biosynthesized ZnO-NPs can be characterized by disruption of cellular structures such as cell walls, membranes, and organelles; restriction of biological macromolecular activity such as proteins or enzymes; prevention of DNA replication; and disruption of the anti-oxidative system via the production of reactive oxygen species (ROS) and/or Zn^2+^-mediated mechanisms [75]. Moreover, the fungicidal activity of the biosynthesized ZnO-NPs could be attributed to the oxidative stress brought on by the action of ROS that are produced as a result of ZnO-NPs [76]. Additionally, it was proposed that ZnONPs treatment would reduce glutathione (GSH) levels by blocking GSH synthesizing enzymes, decreasing the antioxidant capacity in fungal cells, and ultimately causing fungal mortality by the oxidation of intracellular components [77]. Furthermore, the antifungal efficacy of the biogenic ZnO-NPs can be ascribed to the influence of capping biomolecules such as phenolics, alkanes, alkenes, aldehydes, and primary alcohols, which potentially contribute to their fungicidal properties. Our outcomes were coincident with those of a preceding report, which indicated that ZnO-NPs fabricated utilizing *Salix tetrasperma* extract exhibited antifungal efficiency against *C. albicans* of 23.67  ±  0.29 mm [78]. The biogenic ZnO-NPs (100 µg/disk) exhibited a potent antifungal efficiency against *C. albicans* strain with an inhibition zone diameter of 22.56 ± 0.51 mm, which was significantly higher than that of a preceding report, which indicated that ZnO-NPs synthesized utilizing leaves and stem extracts of *Carissa opaca* revealed anticandidal efficiency against *C. albicans* strain, recording an inhibitory zone of 16.0 ± 1 mm in diameter at a ZnO-NP concentration of 10 mg/mL [39]. The minimum inhibitory concentration (MIC) of ZnO-NPs was observed to be 40 µg/mL, while the minimum fungicidal concentration (MFC) was found to be 80 µg/mL against strains of *C. albicans* and *C. tropicalis*. Conversely, the MIC of biogenic ZnO-NPs against the strain of *Candida glabrata* was determined to be 80 µg/mL, whereas the detected MFC value was 160 µg/mL.

### 3.9. Morphological Deformations of Candidal Cells Treated with the Biogenic ZnO-NPs

**Figure 11:**
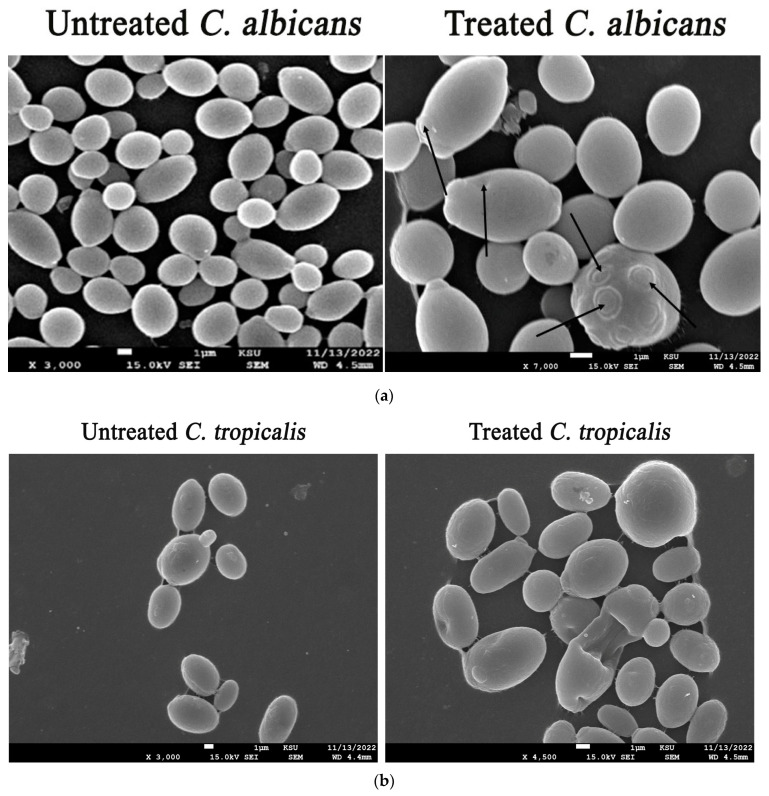
(**a**) Morphological deformations of *C. albicans* treated with ZnO-NPs (the morphological distortions of treated *C. albicans* cells are pointed out by arrows). (**b**) Morphological deformations of *C. tropicalis* treated with ZnO-NPs (the deformations of treated *C. tropicalis* cells are pointed out by arrows).

SEM micrographs of treated *C. albicans* cells reveal the disintegration, deformation, and convolution of the cell walls and membranes, as well as the formation of holes in the cell membrane. Additionally, there have been occasional observations of abnormal infoldings and indentations of the cell wall, as well as loss of membrane integrity (Figure 11a). The presence of cellular debris on the surface of cells is believed to originate from candidal cells that have degenerated. Moreover, the morphological deformations of treated *C. tropicalis* cells were investigated, as seen in Figure 11b. The treated *C. tropicalis* cells become less uniform and exhibit broken cell walls along with destruction of the outer membrane as a result of the development of surface bleb, which also causes the intracellular components to seep out and the nanoparticles to enter, impairing the structural integrity of the cells. Additionally, the formation of holes and pores in the cellular membranes might lead to the leakage of intracellular constituents and, finally, cell death. Our outcomes were in agreement with those of a preceding investigation, which confirmed the development of cell membrane lesions and wrinkles, indicating the fungicidal mode of action of ZnONPs against the treated candidal cells, which ultimately results in fungal cell death [79]. The fungicidal action of the biogenic nanoparticles was observed to be mediated by the generation of reactive oxygen species. This process led to the disruption of the membrane potential, resulting in the formation of pores in the membranes, as evidenced by scanning electron microscopy (SEM) images. Consequently, there was a leakage of intracellular substances and subsequent morphological deformations observed in candidal cells [72]. In addition, SEM images of the treated *C. albicans* cells exhibited the conspicuous presence of multiple pores in the cell wall of the candidal cells. The formation of pores in the candidal cell wall subsequently resulted in the liberation of intracellular constituents and the initiation of apoptosis in the candidal cells.

### 3.10. Synergistic Patterns of the Biogenic ZnO-NPs with Commercial Antifungal Agents

**Figure 12:**
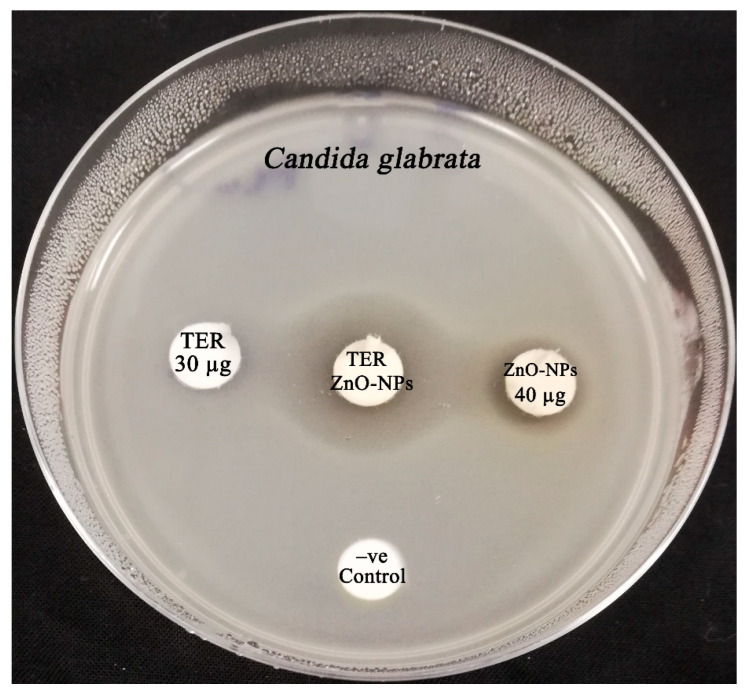
Synergistic antifungal activity of ZnO-NPs formulated using water extracts of *S. officinalis* extract with terbinafine against the *C. glabrata* strain.

The synergistic anticandidal efficiency of ZnO-NPs with commercial antifungal agents such as fluconazole, itraconazole, clotrimazole, nystatin, and terbinafine was evaluated. In this context, the biogenic ZnO-NPs synthesized using water extract of *S. officinalis* leaves revealed the highest synergistic activity with terbinafine antifungal drug against *C. glabrata* strain, recording an IFA value of 6.82, as demonstrated in Table 3 and Figure 12. Table 4 shows the fractional inhibitory concentration index (FICI) of the combined action of biogenic ZnO-NPs and antifungal agents against the tested candidal strains. In the present study, synergistic activity was observed against the *C. albicans* strain for the combination of ZnO-NPs and fluconazole, with a corresponding FICI value of 0.38. Moreover, an additive effect was observed for both the combinations of ZnO-NPs with clotrimazole and ZnO-NPs with nystatin against the *C. albicans* strain, with a corresponding FICI value of 0.75. Interestingly, significant synergistic efficiency was detected for the combination of ZnO-NPs with terbinafine against the *C. glabrata* strain, with a relative FICI value of 0.25. On the other hand, no effect was detected for the combinations of ZnO-NPs with fluconazole and ZnO-NPs with clotrimazole, with relative FICI values of 1.5 and 1.25, respectively. Synergistic anticandidal efficiency was detected for the combination of ZnO-NPs with nystatin against *C. glabrata* and *C. tropicalis*, demonstrating relative FICI values of 0.38 and 0.50, respectively.

The synergistic mode of action might be attributed to the dual action of both terbinafine and ZnO-NPs against different cellular targets. Terbinafine is classified as an allyl amine and functions by targeting ergosterol, which disrupts the synthesis of the cell membrane. This disruption leads to a decrease in cellular permeability, allowing for the internalization of biogenic ZnO-NPs [80]. The fungicidal action of these nanoparticles takes place through the generation of ROS, which oxidize various cellular constituents such as lipids, proteins, and DNA. Ultimately, this oxidative stress leads to cellular death [81]. Moreover, a synergistic pattern of ZnO-NPs with fluconazole antifungal agents was detected against the *C. albicans* strain, with an IFA value of 1.63. The hypothesized mode of synergistic action between both fluconazole and biogenic ZnO-NPs was due to their distinct targeting of fungal cellular constituents. Fluconazole disrupts the activity of the 14-α demethylase enzyme, which is crucial for the conversion of lanosterol to ergosterol, a vital constituent of the fungal cell membrane [82]. This leads to the suppression of ergosterol production, heightened cellular permeability, and eventual release of the fungal cellular components [83]. Conversely, ZnO-NPs have the ability to oxidize crucial cellular components of candidal cells owing to the action of ROS, including DNA and proteins. This process ultimately leads to the initiation of cell death in the fungus [84]. In addition, synergistic anticandidal effectiveness of ZnO-NPs with nystatin antifungal agent was observed against *C. tropicalis* and *C. glabrata* stains, with relative IFA values of 1.06 and 2.39, respectively. Polyene antifungal drugs, including nystatin, are characterized by their elongated chemical structures, which enable them to create channels within the fungal cell membrane. This, in turn, disrupts membrane permeability and results in the release of vital components [85]. The onset of enzymatic inactivity and cellular acidification in fungi is triggered by the influx of hydrogen ions and the outflow of potassium ions from the fungal cell. Moreover, the release of carbohydrates and amino acids from the cells ultimately results in the manifestation of fungal cell death [86]. On the other hand, the antifungal properties of ZnO-NPs against candidal cells are attributed to the production of ROS, which interfere with the oxidative system of the fungal cell and oxidize crucial cellular components, including proteins, DNA, and enzymes [87]. This ultimately hinders the cellular enzymatic system, inhibits cellular replication, and triggers cell death [88]. The supposed mechanism of synergistic action between ZnO-NPs and nystatin antifungal agent could be due to the fact that nystatin decreased membrane permeability, enabling ZnO-NPs to penetrate the cell and interfere with DNA, proteins, and enzymes by generating ROS, culminating in the demise of the fungal cell [89].

### 3.11. Cytotoxicity Assay

**Figure 13:**
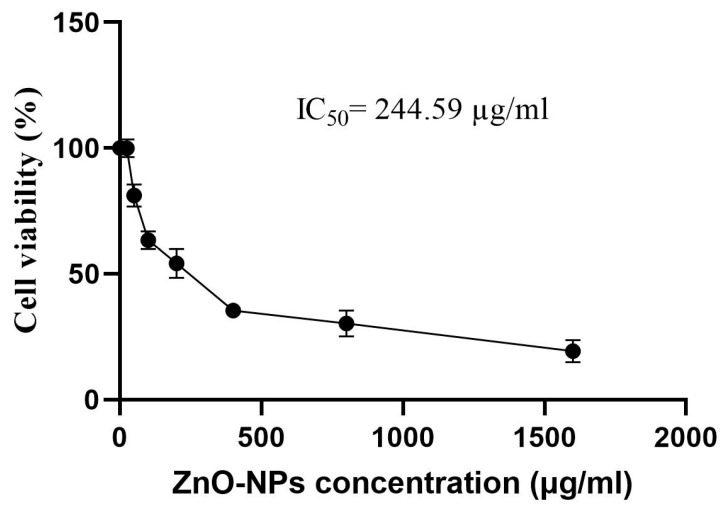
IC_50_ value of the biosynthesized ZnO-NPs against normal lung fibroblast cells.

The cytotoxicity assay was conducted against normal lung fibroblasts to confirm their biosafety for application. In this context, the biogenic ZnO-NPs revealed low cytotoxicity, demonstrating a relative IC_50_ value of 244.59 µg/mL (Figure 13). The low concentration detected affirmed their safety and biocompatibility. The cytotoxicity of the biogenic ZnO nanoparticles exhibited a dependence on concentration, whereby an increase in ZnO concentration resulted in a decrease in cell viability. These values were found to be within the safe limits established by the U.S. National Cancer Institute (NCI), which considers a compound to be toxic if its IC_50_ value is less than 20 µg/mL in the preliminary assays [90]. The cytotoxicity results were consistent with those of a prior investigation, which reported an IC_50_ value of 238 µg/mL for Vero cells after a 72 h incubation period with ZnO-NPs synthesized using *Pichia kudriavzevii* extract as a green method [40]. A previous study reported a relatively low IC_50_ value for the phytosynthesized ZnO-NPs against WI-38 cells, with an average IC_50_ of 642.21 μg/mL [84]. According to the US Food and Drug Administration (FDA), it is recommended that zinc oxide nanoparticles be regarded as safe, specifically falling under the category of generally recognized as safe (GRAS) [91,92]. Overall, the cytotoxicity findings affirmed the safety of the phytosynthesized ZnO-NPs for application.

## 4. Conclusions

The water extract of *S. officinalis* leaves mediated the green biofabrication of ZnO-NPs with potential physicochemical characteristics. The biosynthesized ZnO-NPs revealed potential antifungal effectiveness against the tested candidal pathogens. Moreover, the biogenic ZnO-NPs revealed the highest synergistic effectiveness with terbinafine antifungal agent against the *C. glabrata* strain, whereas the highest synergistic efficiency of ZnO-NPs was detected with fluconazole and nystatin against the tested *C. albicans* and *C. tropicalis* strains, respectively. The high synergistic efficacy of the biogenic ZnO-NPs in combination with antifungal agents such as terbinafine, nystatin, and fluconazole highlights the potential application of these combinations in developing innovative antifungal agents that can boost the antifungal bioactivity of conventional antifungal drugs and address the prevalent issue of fungal resistance to these drugs.

## Figures and Tables

**Table 1:** FTIR groups of ZnO NPs synthesized utilizing sage water extract.

No.	Absorption Peak (cm^−1^)	Appearance	Functional Groups	Molecular Motion
1	3434.86	Strong	Phenolics	O-H stretching
2	2921.47	Medium	Alkanes	C-H stretching
3	1630.70	Medium	Alkenes	C=C stretching
4	1386.59	Weak	Aldehydes	C-H bending
5	1076.15	Medium	Primary alcohols	C-O stretching
6	576.06	Weak, broad	Metal oxide bonds	Zn-O stretching

**Table 2:** Antifungal efficiency of the greenly synthesized ZnO-NPs against candidal pathogens.

Candidal Strains	Inhibition Zone Diameters (mm)			
	ZnO-NPs (50 μg/disk)	ZnO-NPs (100 μg/disk)	Terbinafine (30 µg/disk)	Negative Control
*C. albicans*	18.27 ± 0.16 ^a^	22.56 ± 0.51 ^a^	25.73 ± 0.27 ^a^	0.00 ± 0.00 ^a^
*C. glabrata*	13.53 ± 0.64 ^b^	15.12 ± 0.38 ^b^	8.54 ± 0.21 ^b^	0.00 ± 0.00 ^a^
*C. tropicalis*	19.68 ± 0.32 ^a^	23.17 ± 0.45 ^a^	34.82 ± 0.12 ^c^	0.00 ± 0.00 ^a^

Means followed by different superscript letters in each column differ significantly at *p* ≤ 0.05.

**Table 3:** Increase in fold of inhibition area (IFA) of the combined ZnONPs + antifungals compared to antifungals only.

Concentrations (µg/disk)	Inhibition Zone Diameter (mm)		
	*C. albicans* (IFA)	*C. glabrata* (IFA)	*C. tropicalis* (IFA)
CLO (10 µg)	16.11 ± 0.63	26.15 ± 0.38	32.19 ± 0.53
CLO (10 µg) + ZnONPs (10 µg)	19.57 ± 0.42 (0.48) *	23.14 ± 0.45 (−0.22) *	27.15 ± 0.24 (−0.41) *
FLU (25 µg)	8.97 ± 0.54	23.49 ± 0.21	30.19 ± 0.12
FLU (25 µg) + ZnONPs (10 µg)	14.57 ± 0.12 (1.63) *	23.87 ± 0.23 (0.03) ^ns^	32.84 ± 0.56 (0.81) *
ITZ (10 µg)	20.68 ± 0.18	11.98 ± 0.52	25.12 ± 0.37
ITZ (10 µg) + ZnONPs (10 µg)	21.39 ± 0.56 (0.07) ^ns^	15.96 ± 0.83 (0.77) *	23.16 ± 0.43 (−0.18) *
NST (25 µg)	15.33 ± 0.19	8.76 ± 0.54	9.13 ± 0.23
NST (25 µg) + ZnONPs (10 µg)	20.12 ± 0.17 (0.72) *	16.14 ± 0.29 (2.39) *	13.12 ± 0.34 (1.06) *
TER (30 µg)	27.12 ± 0.56	8.34 ± 0.42	36.14 ± 0.29
TER (30 µg) + ZnONPs (10 µg)	29.17 ± 0.21 (0.16) *	24.16 ± 0.54 (6.82) *	37.15 ± 0.42 (0.06) ^ns^

* Asterisks indicate that values were significantly different compared to control (antifungal agents) at *p* ≤ 0.05. The abbreviation (ns) indicates that values were not significantly different compared to the control (*p* > 0.05).

**Table 4:** The fractional inhibitory concentration index (FICI) of the combined action of biogenic ZnO-NPs and antifungal agents.

Candidal Strains	Combined ZnO+ Antifungal Agent	FICI	Action
*C. albicans*	ZnO-NPs + clotrimazole	0.75	Additive
	ZnO-NPs + fluconazole	0.38	Synergistic
	ZnO-NPs + itraconazole	1.50	No effect
	ZnO-NPs + nystatin	0.75	Additive
	ZnO-NPs + terbinafine	1.25	No effect
*C. glabrata*	ZnO-NPs + clotrimazole	1.25	No effect
	ZnO-NPs + fluconazole	1.50	No effect
	ZnO-NPs + itraconazole	0.63	Additive
	ZnO-NPs + nystatin	0.38	Synergistic
	ZnO-NPs + terbinafine	0.25	Synergistic
*C. tropicalis*	ZnO-NPs + clotrimazole	1.50	No effect
	ZnO-NPs + fluconazole	1.00	Additive
	ZnO-NPs + itraconazole	2.00	No effect
	ZnO-NPs + nystatin	0.50	Synergistic
	ZnO-NPs + terbinafine	1.50	No effect

Synergistic effect ≤ 0.5; additive 0.5 to 1; no effect >1 to 4 and antagonistic >4.

## Data Availability

Not applicable.
